# Unaltered expression of the lncRNA NRON in lesional skin from patients with psoriasis

**DOI:** 10.1515/rir-2026-0030

**Published:** 2026-10-22

**Authors:** Gabriela Fonseca-Camarillo, Carlos A Barrera-Ochoa, Yaneli Juárez-Vicuña, María Elisa Vega-Memije, Rashidi Springall, Jesús K Yamamoto-Furusho, Luis M Amezcua-Guerra

**Affiliations:** Immunology Department, Instituto Nacional de Cardiología Ignacio Chávez, Mexico City, Mexico; Dermatology Department, Hospital General “Dr. Manuel Gea González”, Mexico City, Mexico; Inflammatory Bowel Disease Clinic, Department of Gastroenterology, Instituto Nacional de Ciencias Médicas y Nutrición Salvador Zubirán, Mexico City, Mexico; Health Care Department, Universidad Autónoma Metropolitana–Xochimilco, Mexico City, Mexico; School of Medicine, Universidad La Salle, Mexico City, Mexico

Dear Editor,

Psoriasis is a chronic immune-mediated skin disease characterized by dysregulated immune cell infiltration and aberrant keratinocyte proliferation. Its pathogenesis involves multiple inflammatory pathways, including activation of the nuclear factor of activated T cells (NFAT) downstream of calcineurin signaling, which has informed the development of targeted therapies such as tumor necrosis factor (TNF) inhibitors (*e.g*., adalimumab) and calcineurin inhibitors (*e.g*., tacrolimus).^[1]^ However, these therapies are not curative, underscoring the need to identify additional molecular mechanisms involved in disease regulation.

Epigenetic regulators, particularly long noncoding RNAs (lncRNAs), have emerged as important contributors to psoriasis pathobiology.^[2]^ Among these, the lncRNA NRON (noncoding repressor of NFAT) functions as part of a ribonucleoprotein complex that retains phosphorylated NFAT in the cytoplasm, thereby preventing its nuclear translocation and attenuating NFAT-dependent cytokine expression, including TNF and interleukin-2 (IL-2).^[3,4]^ Altered NRON expression has been reported in autoimmune diseases such as Sjögren’s disease and rheumatoid arthritis; however, its relevance in psoriasis remains unclear.^[2,5,6]^ In this context, we investigated whether NRON transcript abundance is altered in psoriatic lesional skin.

We analyzed stored RNA from 21 patients with plaque psoriasis who were not receiving systemic therapy at the time of biopsy and from 14 control skin samples obtained during flap excision procedures.^[7]^ The psoriasis group included 10 women (median age, 51 years; interquartile range [IQR], 47–61]), whereas the control group included 9 women (median age, 38 years; IQR, 34–66). Disease severity was assessed using the Psoriasis Area and Severity Index (PASI; median, 8.8; IQR, 5.4–19.9) and body surface area involvement (median, 10%; IQR, 4–27.5). NRON (ENST00000517270) expression was quantified by quantitative reverse-transcription polymerase chain reaction (qRT-PCR), normalized to GAPDH (NM_002046) using the 2^ΔΔCt^ method, scaled by 1000, and expressed as arbitrary units.

The study was approved by the local Ethics and Research Committee (No. 06–143–20) and conducted per the Declaration of Helsinki. Written informed consent was obtained from all participants.

NRON transcript levels did not differ significantly between psoriatic lesional skin and control skin (median, 2.00; IQR, 0.80–12.91 *vs*. median, 4.08; IQR, 1.09–23.20; *P* = 0.629). Expression levels exhibited substantial inter-individual variability in both groups. Moreover, NRON expression did not correlate with PASI scores (Spearman’s rho = 0.01; 95% CI, -0.42 to 0.45; *P* = 0.939), or with body surface area involvement (Spearman’s rho = 0.03; 95% CI, -0.41 to 0.47; *P* = 0.864) (Figure 1).

**Figure 1:**
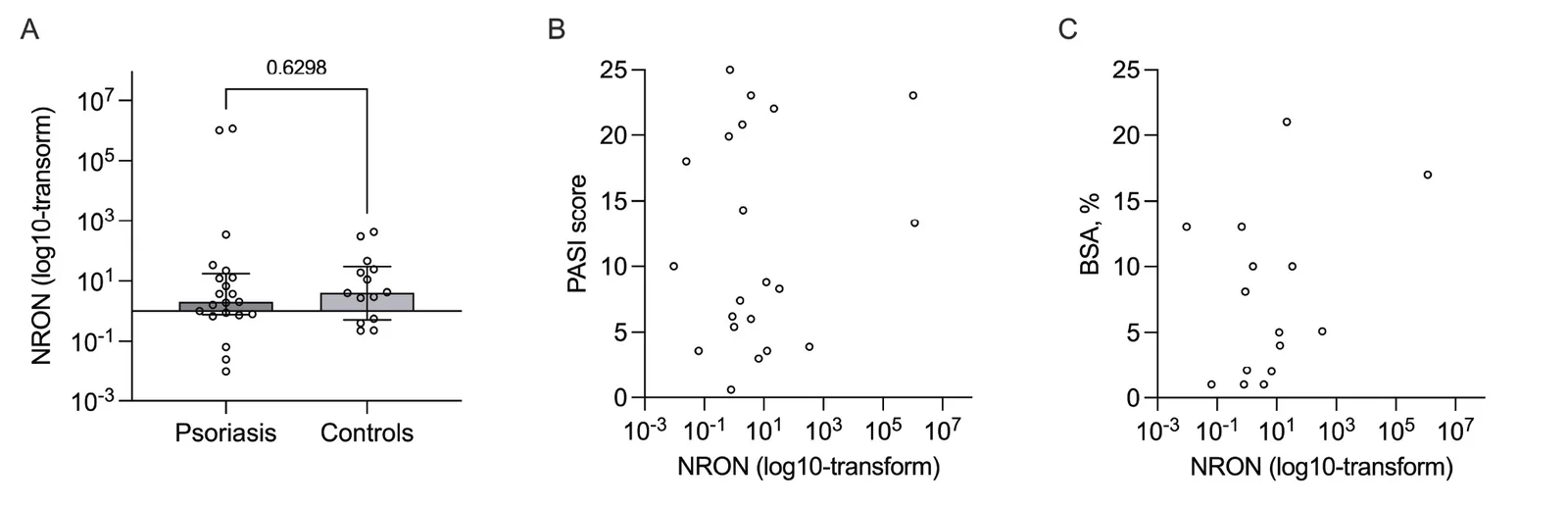
NRON expression in psoriatic lesional skin and its relationship with disease severity. (A) Log10-transformed NRON transcript levels in lesional skin from patients with psoriasis (n = 21) and control skin samples (n = 14). Bars represent median values with interquartile ranges. Differences between groups were assessed using the Mann–Whitney U test. (B) Correlation between NRON expression and Psoriasis Area and Severity Index (PASI) scores (Spearman’s rho = 0.01; 95% CI from-0.42 to 0.45; P = 0.939) in patients with psoriasis. (C) Correlation between NRON expression and body surface area (BSA) involvement (Spearman’s rho = 0.03; 95% CI from-0.41 to 0.47; P = 0.864).

These findings suggest that steady-state NRON transcript abundance is not significantly altered in bulk lesional skin from patients with psoriasis. This negative result should, however, be interpreted with caution. We did not assess NFAT activation, downstream cytokine expression, or the functional consequences of NRON modulation. In addition, control samples were obtained during flap excision procedures, and the control group was younger than the psoriasis cohort, which may introduce selection bias.

To further contextualize our findings, we interrogated publicly available single-cell transcriptomic datasets of psoriatic skin (https://sccamel.readthedocs.io/andhttps://yz-studio.shinyapps.io/psoriaticskincellatlas2/). These analyses indicate that gene expression is highly compartmentalized across keratinocytes, fibroblasts, and immune cell subsets.^[8,9]^ Consistent with this framework, NRON does not emerge as a prominently differentially expressed lncRNA across major skin cell populations, supporting our observation of unchanged bulk expression. In contrast, members of the NFAT family are predominantly expressed in CD4^+^ and CD8^+^ T cells, with limited and heterogeneous expression in dendritic cells and keratinocytes. Given that NFAT activity is primarily regulated post-translationally through calcineurin-dependent dephosphorylation and nuclear translocation, transcriptomic data alone may not fully capture pathway activation.

Collectively, these observations suggest that NRON may exert context-dependent effects within specific cellular subsets or through post-transcriptional mechanisms not captured by bulk RNA quantification. Moreover, lncRNA function frequently depends on interactions with chromatin and protein complexes–the so-called “lncRNA interactome”–which cannot be inferred solely from transcript abundance.^[10]^ These limitations are particularly relevant given the modest sample size and exploratory nature of the study.

In conclusion, bulk qRT-PCR analysis did not reveal differential NRON expression in psoriatic lesions. Future studies integrating single-cell transcriptomics with functional assays will be required to determine whether NRON contributes to psoriasis pathogenesis in a cell type-specific manner.
